# Endogenous TCR Recombination in TCR Tg Single RAG-Deficient Mice Uncovered by Robust *In Vivo* T Cell Activation and Selection

**DOI:** 10.1371/journal.pone.0010238

**Published:** 2010-04-29

**Authors:** Caroline Montaudouin, Laurent Boucontet, Marie-Pierre Mailhé-Lembezat, Maria-Encarnita Mariotti-Ferrandiz, Anne Louise, Adrien Six, Antonio A. Freitas, Sylvie Garcia

**Affiliations:** 1 Unité de Biologie des Populations Lymphocytaires, Département d'Immunologie, Institut Pasteur, Centre National de Recherche Scientifique-Unité de Recherche Associée 1961, Paris, France; 2 Unité du Développement des Lymphocytes, Département d'Immunologie, Institut Pasteur, Institut National de la Santé et de la Recherche Médicale U668, Paris, France; 3 Unité de Physiopathologie des Infections, Département d'Immunologie, Institut Pasteur, Centre National de Recherche Scientifique-Unité de Recherche Associée 1961, Paris, France; 4 Plate-forme de Cytométrie, Département d'Immunologie, Institut Pasteur, Paris, France; INSERM U768, Pavillon Kirmisson, France

## Abstract

Recombination activating gene (RAG)-deficient TCR (T Cell Receptor) Tg (transgenic) mice are routinely used as sources of monoclonal T cells. We found that after the transfer of T cells from a RAG-2-deficient 5CC7 TCR Tg mice into allogeneic hosts we recovered a population of T cells expressing diverse αβ-TCRs. In fact, in the thymus and spleen of the 5CC7 RAG-2-deficient donor mice, we detected rare T cells expressing non-Tg TCR chains. Similar observations were obtained using T cells from two other TCR transgenic strains, namely RAG-2-deficient aHY and RAG-1-deficient OT-1 mice. The sequences of the endogenous TCR transcripts suggested that gene recombination could occur, albeit quite inefficiently, in the RAG-deficient mice we used. In agreement, we evidenced rare TCR Vα and Vβ-chain transcripts in non-Tg RAG-2-deficient mice. Since in these non-Tg RAG-deficient mice no mature T cells could ever be found, our findings suggested a role for the TCR Tg in rescuing rare recombined endogenous chains. Robust T-cell activation by the allogeneic environment favored the selection and expansion of the rare cells expressing endogenous TCRs. Potential mechanisms involved in the recombination of the endogenous TCR chains in the different strains of RAG-deficient mice used, and in particular the possibility of RAG-1 hypomorphism due to an incomplete knocking out procedure, are discussed. Our findings have important experimental implications for studies using TCR-Tg RAG-deficient cells as monoclonal T cell populations.

## Introduction

The development of T cell receptor (TCR) transgenic (Tg) mice offered a promising tool to circumvent the low frequency of T-cells specific for a given antigen [1], [2]. Indeed, these mice permitted valuable studies on T cell development and immune responses [1], [2], [3]. However, endogenous TCR expression was still observed [4], reflecting mainly the incomplete allelic exclusion of the TCRα chain. To obtain pure monoclonal T cell populations, TCR Tg mice were crossed with RAG-1 or RAG-2-deficient mice [5], [6], [7]. The lymphocyte-specific recombination genes *RAG-1* and *RAG-2* encode RAG-1 and RAG-2 proteins that together form a complex responsible for recognizing and cutting V, D and J segments thereby initiating V(D)J rearrangement [8]. Since it was understood that recombination requires both *RAG* genes [9], the functional impairment of only one of the two genes was believed to abolish any endogenous TCR or B cell receptor (BCR) expression. In agreement it was found that either RAG-1 or RAG-2-knocked out mice have no detectable T and B cells [5], [6], [7] and when crossed into a TCR Tg background, they appeared to contain a single homogeneous monoclonal population of mature T-cells expressing the TCR-Tg and no B cells [10].

We exploited this property to study the fate of monoclonal CD4 naïve T-cells in different MHC environments. We found that upon transfer into allogeneic RAG^0/0^ γc^0/0^ hosts, T cells from TCR Tg RAG-2-deficient mice, namely the 5CC7 strain, proliferate. However, we unexpectedly found that with time most of the donor T cells recovered from the allogeneic hosts did not express the TCR Tg, but expressed other endogenous αβ TCRs. Based on these observations, we were able to detect rare T cells expressing non-Tg TCRs in the thymus and periphery of the donor mice in spite of their RAG-deficiency. Sequence analysis of the expressed endogenous TCRs strongly suggested that RAG-dependent TCR recombination occured in the RAG-knocked out (KO) strains used. Similar observations were obtained using aHY TCR Tg RAG-2-and OT-1 TCR Tg RAG-1 deficient strains. If in the case of the RAG-2-deficient mice it is conceivable that RAG-1 alone could perform VDJ recombination, this hypothesis is very unlikely for RAG-1-deficient mice. However, two RAG-1 knockout alleles have been generated and the RAG-1 KO strain we have analyzed here has the potential to be a hypomorphic allele due to the remaining expression of the essential catalytic RAG-1 core.

## Results

### Expression of endogenous TCR-chains by T cells from TCR Tg RAG-deficient mice transferred into allogeneic hosts

**Figure 1:**
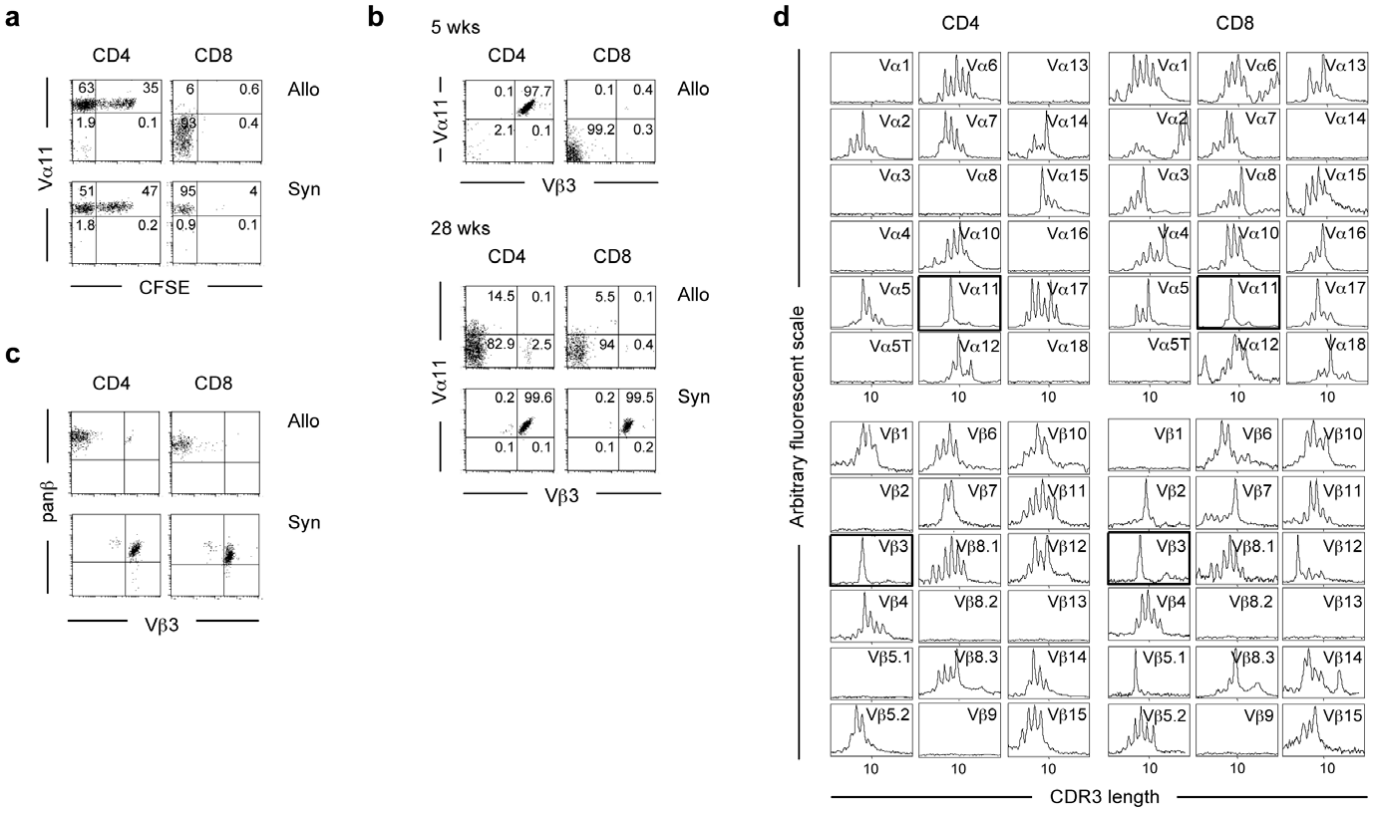
Emergence of non Tg TCR expressing T-cells after transfer into allogeneic hosts. One million CFSE-labeled LN T-cells from 5CC7 RAG-2-deficient mice were injected into syngeneic H-2^a^ or allogeneic H-2^b^ RAG-2^0/0^γc^0/0^ hosts. Five (a,b) and 28 (b,c and d) weeks later, mice were sacrificed and spleen cells were analyzed by Flow Cytometry or Immunoscope. (**a**) Dot plots show CFSE dilution and Vα11 TCR Tg chain expression by gated CD3^+^CD4^+^ or CD3^+^CD8^+^ 5 weeks after transfer. (**b**) Dot plots show expression of Vα11 and Vβ3 TCR Tg chains by gated CD3^+^CD4^+^ or CD3^+^CD8^+^ at 5 and 28 weeks after transfer; (**c**) Dot plots show that 28 weeks after transfer into allogeneic hosts both CD3^+^CD4^+^ and CD3^+^CD8^+^ Vβ3^−^ T-cells were panβ^+^, i.e. they expressed a αβ TCR. Each dot plot is representative of 3 to 6 different mice (**d**) Twenty-eight weeks after transfer, mRNA was extracted from CD4 or CD8 donor T-cells recovered from pooled allogeneic hosts. mRNA were retro-transcribed and amplified using several Vα (top panels) and Vβ (bottom panels) specific primers. CDR3 length profiles obtained by Immunoscope are shown, with CDR3 size on x-axis (peaks are separated by 1 amino acid), and arbitrary scale of fluorescence on y-axis (proportional to the RT-PCR product amount). The bold framed profiles correspond to the Vα11 or Vβ3 Tg transcripts.

To compare the fate of monoclonal TCR Tg 5CC7 T cells in different MHC environments, we transferred CFSE-labeled T-cells from H-2^a^ 5CC7 TCR Tg RAG-2-deficient [6] donors into either H-2^a^ (syngeneic) or H-2^b^ (allogeneic) RAG-2^0/0^γc^0/0^ hosts. Deprived of T, B and NK cells, these hosts are unable to reject allogeneic donor cells. We studied CFSE-dilution and expression of the TCR Vα11 and Vβ3 Tg chains by the donor T-cells. Five weeks after transfer, the majority of the T-cells divided in both syngeneic and allogeneic hosts (Figure 1a). Unexpectedly, while in the syngeneic hosts 80% of the recovered T-cells were CD4^+^ and Vα11^+^, in the allogeneic hosts 40% were CD8^+^ T-cells and Vα11^−^ (Figure 1a). The study of the co-expression of Vα11 and Vβ3 TCR Tg chains confirmed that in the allogeneic hosts most of the CD4^+^ T-cells were Vα11^+^ Vβ3^+^ (Figure 1b), but only a minority (0.4%) of the CD8^+^ T-cells expressed the TCR αβ Tg chains. Moreover, at 28 weeks after transfer, while in the syngeneic hosts virtually all donor T-cells remained Vα11^+^Vβ3^+^, almost none of the CD4^+^ and CD8^+^ T-cells recovered in the allogeneic hosts expressed the TCR Tg chains (Figure 1b). Noteworthy, all the CD3^+^ TCR Tg T-cells recovered from allogeneic hosts expressed a TCRβ chain (Figure 1c) and were TCR γδ^−^ (data not shown), confirming that these Vα11^−^Vβ3^−^ cells expressed a TCR and belonged to the αβ T cell lineage.

In order to confirm the expression of endogenous TCRs by the donor RAG-2-deficient T-cells in the allogeneic hosts, we analyzed their TCRα and β chain usage by Immunoscope [11]. We found that CD4 and CD8 T-cell populations expressed multiple non-Tg α and β TCR-chain transcripts with significant Complementaring Determining Region (CDR)3 length complexity (Figure 1d). Interestingly, we were unable to detect Vα11- and Vβ3-peaks other than those corresponding to the Tg-encoded transcripts. This observation suggests the presence of dual TCR expressing T-cells where the abundant Tg chain message may have masked the detection of the endogenous rearranged Vα11- and Vβ3-transcripts. The finding of Vβ3-Cβ transcripts expressing Jβ gene segments different from the Jβ Tg confirmed this hypothesis (not shown).

It is important to note that we confirmed the RAG-deficiency in all donor and host mice both by Flow Cytometry and by a 35–40 cycle PCR (using specific RAG-1 and RAG-2 primers able to distinct between WT and KO allele) (see M&M). We should add that by using the same PCR and Immunoscope conditions, we were unable to detect any TCRβ mRNA transcripts among the spleen cells of TCRβ gene enhancer^0/0^ mice [12] (not shown).

Taken together, these findings indicate that transfer of TCR-Tg T cells from 5CC7 RAG-2-deficient donors into allogeneic hosts, resulted in the “selection” of donor T cells expressing diverse endogenous TCRs.

### Endogenous TCR expression in the thymus and periphery of TCR Tg RAG-deficient mice

**Figure 2:**
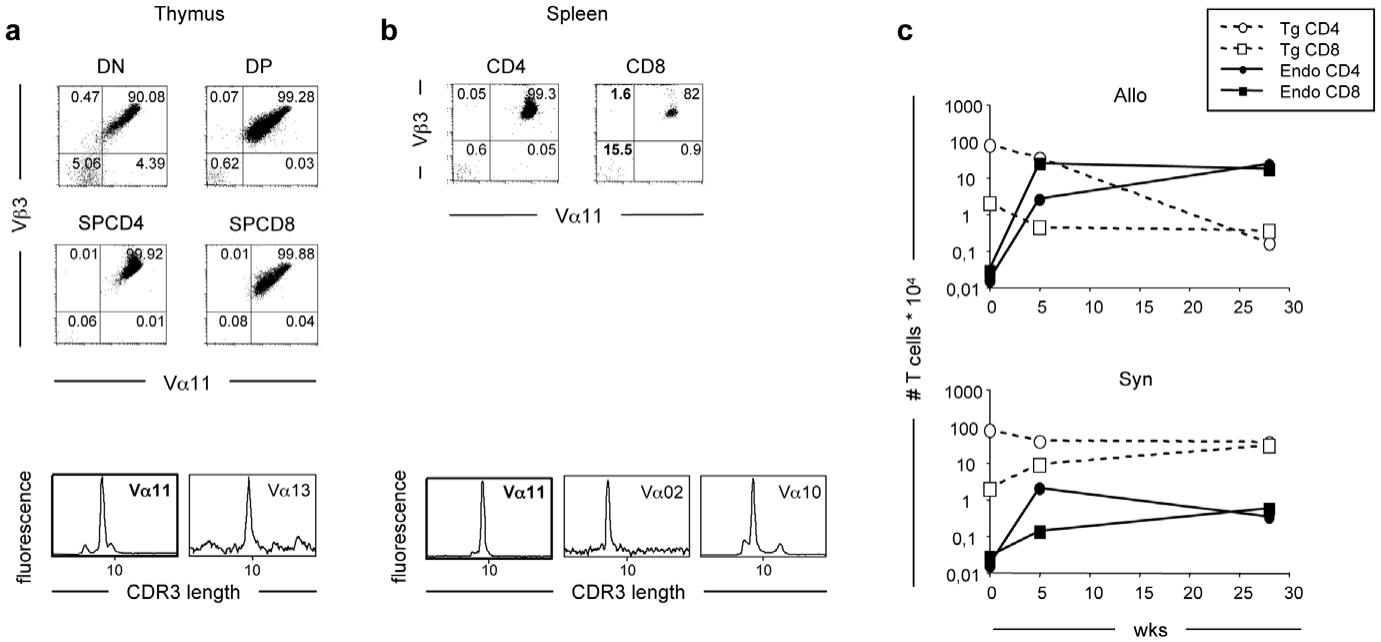
Pre-existence of endogenous TCR expression in the thymus and peripheral lymphoid tissues of 5CC7 RAG-2^0/0^ mice. (**a–b**) Thymus and peripheral cells from 5CC7 RAG-2^0/0^ mice were analyzed for the presence of non-Tg TCR expression both by Flow Cytometry and Immunoscope analyses. (**a**) Top dot plots show the expression of the Vα11 and Vβ3 TCR Tg chains by CD3^lo/hi^DN (CD4^−^CD8^−^), DP (CD4^+^CD8^+^), SPCD4^+^ and SPCD8^+^ thymocytes. Lower panels show CDR3 length profiles for the Vα11 Tg and other non-Tg Vα transcripts present in retro-transcribed and amplified mRNA from total thymocytes using several Vα specific primers. (**b**) Top dot plots show the expression of the Vα11 and Vβ3 TCR Tg chains by CD3^+^CD4^+^ or CD3^+^CD8^+^ spleen T-cells. Bottom panels show CDR3 length profiles for the Vα11 Tg and other non-Tg Vα transcripts present in retro-transcribed and amplified mRNA from total spleen cells using several Vα specific primers. (**c**) T-cells from 5CC7 RAG-deficient donors were transferred into allogeneic or syngeneic hosts. Panels show the numbers of endogenous (filled line) and Tg (dotted line) TCR expressing CD4^+^ (circles) or CD8^+^ (squares) T-cells present in the inoculum and 5 and 28 weeks after injection in allogeneic (upper graph) and syngeneic (lower graph) hosts.

Endogenous TCR chain expression by the T-cells recovered from the allogeneic hosts could result from the expansion of pre-existing donor T cells or be “de novo” induced upon T cell transfer. We investigated whether rare T-cells expressing endogenous TCRs pre-existed in the 5CC7 RAG-2^0/0^ donors. Surprisingly and in spite of the RAG-2-deficiency, we detected expression of non-Tg Vα and Vβ TCRs in the thymus and periphery of the donor mice (Figure 2). On average from 100×10^6^ thymocytes analyzed, 0.5–1% of the double positive (DP) CD3^+^ thymocytes (about 10000 DP cells) and about 0.1% of the single positive (SP) T-cells (30000 SPCD4 and 1500 SPCD8) did not express one or both TCR Tg chains (Figure 2a upper panels). These observations were confirmed by Immunoscope, which revealed the presence of rare non-Tg TCRα chain transcripts among the thymus cells (Figure 2a lower panels). Peripheral CD8^+^ T-cells from 5CC7 RAG-2^0/0^ were more prone to express endogenous TCRs than the CD4^+^ T-cells (Figure 2b upper panels). The expression of endogenous TCRs by the CD8 T cells varied however, from 0.1% to 15% in different individual mice (not shown). We also detected endogenous Vα2 and Vα10 transcripts in the spleen of the 5CC7 RAG-2^0/0^ donor mice (Figure 2b lower panels). These results strongly suggest the preexistence of endogenous TCR bearing T cells in the 5CC7 RAG-2^0/0^ mice, among which some may have been selected and expanded in the allogeneic environment.

Based on this observations, we estimated the number of cells expressing Tg and/or endogenous TCR chains present among the transferred T-cells and evaluated their expansion after transfer into allogeneic and syngeneic hosts. In allogeneic hosts, the number of CD4^+^ and CD8^+^ T-cells expressing endogenous TCRs increased dramatically (≥1000 fold) and represented the majority of the T-cells recovered 28 weeks after transfer, while the number of TCR Tg expressing cells decreased with time. In contrast, in syngeneic hosts, T-cells expressing Tg TCRs represented the majority of the recovered T-cells (Figure 2c). These results strongly indicate that the allogeneic environment favors the selection and expansion of pre-existing endogenous TCR bearing T-cells.

### Endogenous TCR diversity evokes RAG-dependent recombination

Endogenous TCR expression in the 5CC7 RAG-2-deficient mice could be mediated either by RAG-dependent recombination [13], gene conversion [14] or gene replacement [15] mechanisms involved in the generation of BCR and TCR diversity. To discriminate between these possibilities, we sequenced some of the TCRα and TCRβ-chains used by T-cells recovered from the allogeneic hosts. The sequences of the endogenous Vα10 and Vβ8.1 TCR chains evidenced the usage of diverse Jα, Dβ and Jβ segments (Table 1). The number of genes involved argues against a gene replacement mechanism, that could only occur if different Tg copies were inserted in proximity to both the TCR-α and TCR-β loci, an unlikely event. The perfect alignment between the V sequences and the corresponding germ line sequences are inconsistent with the insertion of V sequence fragments, a hallmark of gene conversion. The sequences also showed considerable CDR3 length diversity using different D- and J-gene segments and containing motifs reminiscent of N/P additions [16]. Overall our findings suggest that RAG-dependent endogenous TCR recombination occurs in T-cells from 5CC7 donor mice despite their RAG-2-deficiency.

**Table 1:** Vα/Jα and Vβ/Dβ/Jβ sequences of the TCR transcripts expressed by T cells recovered from allogeneic hosts.

time	Vα	*N/P n*	Jα	
**d0 Thymus**	**Vα11** TGT GCT GCT GAG G		CT TCC AAT ACC AAC AAA GTC GTC TTT GGA ACA GGG ACC	Jα34*02
**d0 Spleen**	**Vα11** TGT GCT GCT GAG G		CT TCC AAT ACC AAC AAA GTC GTC TTT GGA ACA GGG ACC	Jα34*02
**28 wks Spleen (CD4^+^)**	**Vα10** TGT GCT ATG GA	*A CGG*	ATG GGC TAC AAA CTT ACC TTC GGG ACA GGA ACA	**Jα09**
	**Vα10** TGT GCT	*GCA AGC C*	CT TCT GGC AGC TGG CAA CTC ATC TTT GGA TCT GGA ACC	**Jα22**
	**Vα10** TGT GCT ATG GA	*C CTT TA*	T AAC ACC AAT ACA GGC AAA TTA ACC TTT GGG ATG GGG ACC	**Jα27**
	**Vα10** TGT GCT ATA GAT C	*GA*	GAC ACA AAT GCT TAC AAA GTC ATC TTT GGA AAA GGG ACA	**Jα30**
	**Vα10** TGT GCT ATA GA	*C CTC AA*	C AAT AAC AGA ATC TTC TTT GGT GAT GGG ACG	**Jα31**
	**Vα10** TGT GCT ATG		TCT GGA GGA AGC AAT GCA AAG CTA ACC TTC GGG AAA GGC ACT	**Jα42**
	**Vα10** TGT GCT ATA G	*GT*	AAT AAC AAC AAT GCC CCA CGA TTT GGA GCG GGA ACC	**Jα43**
	**Vα10** TGT GCT	*T*	TT ACT GGC AGT GGT GGA AAA CTC ACT TTG GGG GCT GGA ACA	**Jα44**
	**Vα10** TGT GCT	*GTG GCC CC*	C ACG GGT TAC CAG AAC TTC TAT TTT GGG AAA GGA ACA	**Jα49**
	**Vα10** TGT GCT ATA GA	*A C*	CT GGA GCT AAC ACT GGA AAG CTC ACG TTT GGA CAC GGC ACC	**Jα52**
**28 wks Spleen (CD8^+^)**	**Vα10** TGT GCT AT	*C TC*	C ATG GGC TAC AAA CTT ACC TTC GGG ACA GGA ACA	**Jα09**
	**Vα10** TGT GCT ATA GAT C	*A*	G AAT TAT AAC CAG GGG AAG CTT ATC TTT GGA CAG GGA ACC	**Jα22**
	**Vα10** TGT GCT ATG GA		G AAT TAT AAC CAG GGG AAG CTT ATC TTT GGA CAG GGA ACC	**Jα23**
	**Vα10** TGT GCT ATA GAT		TCC AAT ACC GAC AAA GTC GTC TTT GGA ACA GGG ACC	**Jα34**
	**Vα10** TGT GCT ATG GA	*A CGG G*	AT ACA GGA GGT GCA GAT AGA CTC ACC TTT GGG AAA GGA ACT	**Jα45**
	**Vα10** TGT GC	*C CCA GGG G*	AC TAT GCA AAC AAG ATG ATC TTT GGC TTG GGA ACC	**Jα47**
	**Vα10** TGT GCT A	*CC CA*	A TCC TCC TCC TTC AGC AAG CTG GTG TTT GGG CAG GGG ACA	**Jα50**
	**Vα10** TGT GCT ATG GA	*A CGG TCC*	GGC ACT GGG TCT AAG CTG TCA TTT GGG AAG GGG GCA	**Jα58**

time	Vβ	*N/P n*	Dβ	*N/P n*		Jβ
**d0 Spleen**	**Vβ3** TGT GCC AGC AG	*T CTG A*	AC A	*AT G*	CA AAC TCC GAC TAC ACC TTC GGC TCA GGG	**Jβ1–2**
**28 wks Spleen (CD4^+^)**	**Vβ8.1** TGT GCC AGC AGT GAT G	*C*	G A	*A*	T TCT GGA AAT ACG CTC TAT TTT GGA GAA GGA	**Jβ1–3**
	**Vβ8.1** TGT GCC AGC AGT GA	*C A*	GG GAC		CAG GCT CCG CTT TTT GGA GAG GGG	**Jβ1–5**
	**Vβ8.1** TGT GCC AGC AGT GAT		AGG	*CAA AAT C*	CA AAC ACC GGG CAG CTC TAC TTT GGT GAA GGC	**Jβ2–2**
	**Vβ8.1** TGT GCC AGC A	*C*	C AGG		ACC GGG CAG CTC TAC TTT GGT GAA GGC	**Jβ2–2**
	**Vβ8.1** TGT GCC AGC A	*TC*	GGA CTG GGG GGG C	*TT*	GAA CAG TAC TTC GGT CCC GGC	**Jβ2–7**
	**Vβ8.1** TGT GCC AGC AGT G	*GG*	AGG G	*AT*	GAA CAG TAC TTC GGT CCC GGC	**Jβ2–7**
	**Vβ8.1** TGT GCC AGC AGT GAT G	*T*	T GGG GGG GC	*G CTC CAT*	GAA CAG TAC TTC GGT CCC GGC	**Jβ2–7**

The PCR products derived from the Immunoscope studies performed at different times after transfer were sequenced using Vα and Cα, and Vβ and Cβ specific primers.

### Endogenous TCR recombination in donor T cells from other TCR Tg RAG-deficient T cells upon transfer into allogeneic hosts

**Figure 3:**
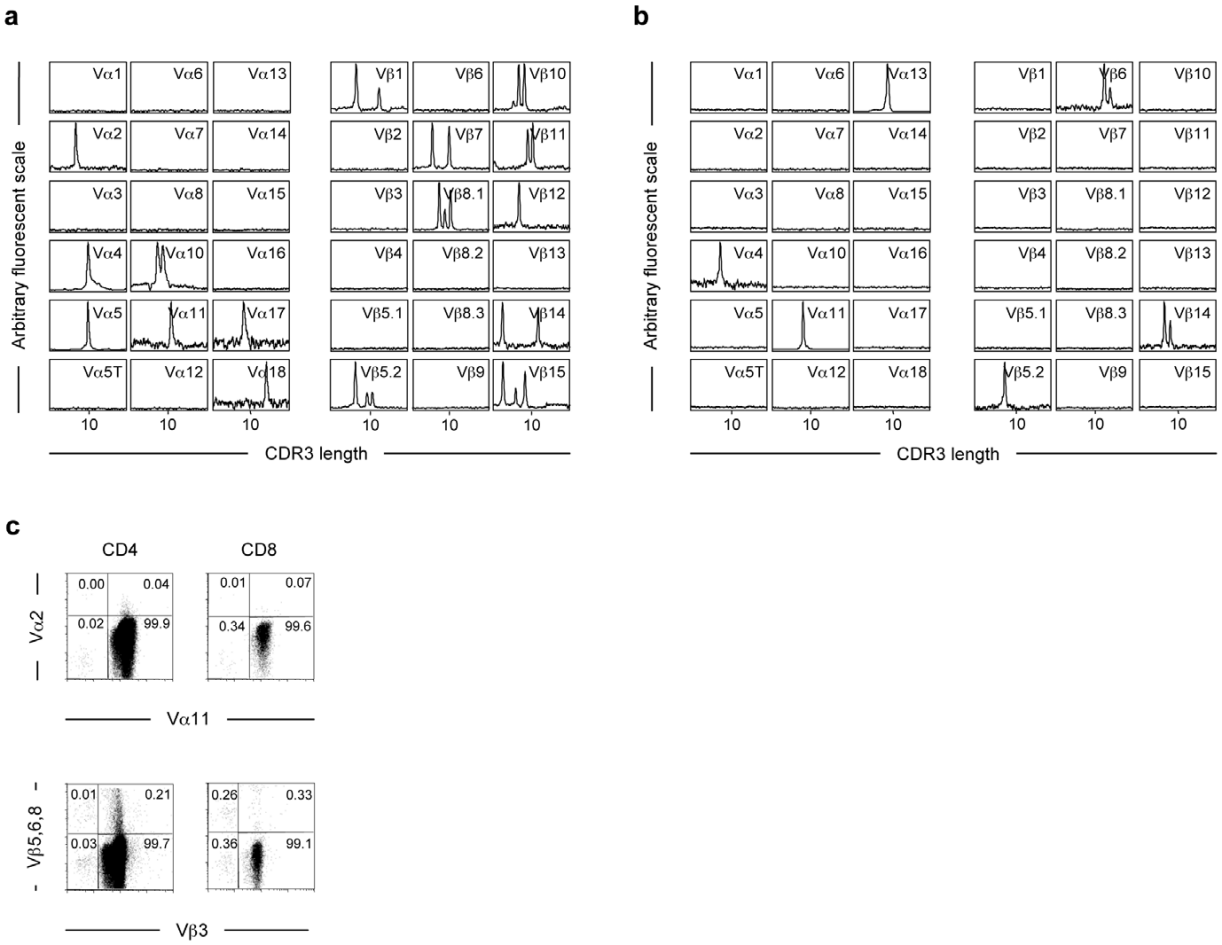
Role of the TCR Tg in endogenous TCR expression. (**a**) Endogenous TCR rearrangements in donor T cells from OT-1 TCR Tg RAG-1- and aHY TCR Tg RAG-2-deficient mice after transfer into allogeneic hosts. One million LN T-cells from OT-1 RAG-1-deficient or aHY RAG-2-deficient mice were transferred into allogeneic RAG-2^0/0^ γc^0/0^ H2^a^ hosts. At 9 (OT-1) and 30 (aHY) weeks after transfer, mice were sacrificed. Total RNA was extracted from spleen cells of each hosts and Immunoscope analyses were performed for Vα chains of aHY donor T cell TCRs (left panel) and Vβ chains of OT-1 donor T cell TCRs (right panel). (**b**) Total mRNA was extracted from the spleen of a RAG-2^0/0^ mouse, retro-transcribed, and amplified using several Vα (left panel) and Vβ (right panel) specific primers. CDR3 length profiles are shown. (**c**) Shows co-expression of Vα11 and Vβ3 TCR Tg chains with the non-Tg Vα2 or Vβ5.1.2, 6 and 8.1.2.3 TCR chains analyzed by flow cytometry on CD3^+^CD4^+^ or CD3^+^CD8^+^ LN T-cells from 5CC7 TCR Tg RAG-2^0/0^ mice.

We next asked whether endogenous TCR recombination could also occur in other TCR Tg RAG-deficient strains. We detected rare non-Tg endogenous TCR chain transcripts after transfer of RAG-2-deficient [6] aHY (Figure 3a, left panel) and RAG-1-deficient [7] OT-1 (Figure 3a, right panel) donor T-cells into allogeneic RAG-2^0/0^γc^0/0^H-2^a^ hosts. These results demonstrate that the RAG-1- or RAG-2-deficient mice we have analyzed and which are currently in use are still able to perform V(D)J TCR recombination and that this process is independent of the specificity and site of insertion of the TCR Tg.

### Role of the TCR Tg in endogenous TCR expression

The absence of T cells in RAG-knocked out mice has been largely reported leading to the assumption that the deficiency for one RAG protein leads to the complete abolishment of TCR recombination. In the light of our present results, we decided to analyze TCR-encoding mRNAs in the spleen of RAG-2-deficient mice, even though there was no evidence of any T cell development in these mice. Using this approach, we were able to evidence some rare TCR Vα- and Vβ-chain transcripts in the spleen cells of RAG-2^0/0^ mice (Figure 3b), probably reflecting recombination that may have occurred in non-T, non-B cells, as reported for NK cells [17]. TCR expression requires, however, productive recombination in the two TCR chain loci. Thus, in the absence of one RAG protein, the probability that such rare recombination events might occur in the two TCR loci and in the same cell must be too low to allow any T cell development. However, in the presence of TCR Tg the rare recombined single TCR chains (that can be detected in non-Tg RAG-deficient mice) could readily associate with one of the Tg chains allowing the protein to reach the cell surface and give rise to new receptor specificities. If this was the case we would expect some 5CC7 T cells to express “dual” TCRs. By flow cytometry, we confirmed the existence of “dual” TCR-expressing T cells in 5CC7 RAG-2^0/0^ mice (Figure 3c). About 0.04% of CD4^+^Vα11^+^ and 0.07% of the CD8^+^Vα11^+^ LN 5CC7 T cells expressed the non-Tg Vα2 chain and 0.21% of CD4^+^Vβ3^+^ and 0.33% of the CD8^+^Vβ3^+^ LN 5CC7 T cells expressed non-Tg TCRβ chains including Vβ5.1, 5.2, Vβ6 or Vβ8.1, 8.2, 8.3 (Figure 3c). We concluded that TCR Tgs play a critical role in resuing rare recombination events occurring in single RAG mice and allowing surface expression of endogenous TCR chains by T cells.

## Discussion

In the present report, we clearly show that endogenous TCR recombination occurs, albeit quite inefficiently, in RAG-2 (or RAG-1) deficient TCR Tg mice. While this observation was first made on 5CC7 TCR Tg RAG-2-deficient mice, it is important to state that T cell populations from two others TCR Tg RAG-deficient strains, the aHY TCR Tg RAG-2-deficient mice, Tg for an H-2D^b^-restricted TCR, and the OT-1 TCR Tg RAG-1-deficient mice, Tg for an H-2K^b^-restricted TCR, also expressed endogenous TCRs after transfer into allogeneic RAG-2^0/0^γc^0/0^H-2^a^. This indicates that endogenous TCR recombination in T cells from TCR Tg RAG-deficient mice is not restricted to a particular TCR Tg strain. The nature of the TCR sequences obtained strongly support RAG-like activity. Therefore our results suggest that in some of the currently available TCR Tg RAG-deficient mice a single RAG protein could induce VDJ recombination. Although it is acceptable that RAG-1 alone could perform VDJ recombination since it has both specific DNA binding domains and catalytic sites for DNA cleavage (in agreement, we revealed the presence of rare isolated mRNA transcripts showing TCR chain rearrangements in the spleen cells of a non-Tg RAG-2 deficient mouse), this hypothesis is very unlikely for RAG-2 since it lacks both DNA binding activity and catalytic activity. It is possible that the *RAG* gene manipulations used to produce the currently available RAG-1-deficient mice could have lead to hypomorphic forms resulting in residual recombination activity. This may occurs in the RAG-1 deficient mice used here, due to a Neo-gene insertion at the position aa330 [7] that could allow residual recombinase activity of the RAG-1 protein. Thus, the present report challenges the traditional view that both RAG-1 and RAG-2 proteins are strictly required to ensure V(D)J recombination, and indicate that RAG-1 alone may be sufficient to induce, although at very low level, some TCR recombination. In these conditions, one can not exclude that the widely accepted monoclonality of RAG-deficient TCR Tg T cells would be due to the suppression of both RAG loci, by cis or trans effects, resulting from the insertion of the *neo* gene, as reported for other targeted mutations [18]. Alternatively, other recombination proteins than RAG may be involved (it is difficult to formally disprove a non RAG-dependent mechanism as it would require creating a double KO mice either by knocking out both genes in ES cells or by crossing *RAG-1* and *RAG-2* KO mice, a highly improbable event due to the physical linkage of the two loci).

While we clearly showed that rare endogenous TCRs could be generated in RAG-1 and RAG-2-deficient TCR Tg mice, there is no evidence for any B or T cell development in the non-Tg RAG-1^0/0^ or RAG-2^0/0^ mice used in the present study, even though we detected very rare TCR rearrangement in the spleen of non-Tg RAG-2-deficient mice (Figure 3b). Therefore one must invoke a specific role for the TCR Tg in the development of those T cells where endogenous recombination occurs. Considering that the endogenous TCR recombinations observed in non-Tg single RAG-deficient mice are extremely rare, the probability that a productive rearrangements occur in both α and β TCR loci and in the same cell is likely too low to result in receptor expression. Most likely, in the presence of the TCR Tg, the rare recombined endogenous single TCR chains present could readily associate with one of the TCR Tg chains allowing the protein to reach the cell surface and give rise to new receptor specificities. Support for this hypothesis was gathered from the observation that 5CC7 T cells express “dual” TCRs. This was demonstrated both by flowcytometry (Figure 3c) and by Immunoscope analysis where the dominant peak profiles obtained for the transcripts of the Vα11- and Vβ3-Tg chains suggested that the 5CC7 Tg was transcribed in the majority of T cells (Figure 1d). We concluded that the allogeneic transfer provided a selective environment favoring the expansion of rare T cells expressing endogenous TCRs present in the original T cell inoculums. The kinetics of expansion of endogenous bearing T cells in allogeneic hosts strongly supported this hypothesis. The robust T cell activation lead to the down-regulation of the TCR Tg resulting in cells expressing mainly endogenous TCRs. Using dual-TCR Tg mice, it was shown that chronic activation induces similarly the selection of cells expressing preferentially only “one” of the several possible TCRs [19].

Our current findings have important experimental implications. TCR Tg mice have been crossed with RAG-1 or RAG-2-KO mice to obtain single homogeneous monoclonal populations of mature T-cells. We now show that this is not always the case. Several hypotheses can be proposed to explain why we happened upon our unexpected observations while copious other studies using TCR Tg RAG-deficient mice did not. First, rare endogenous rearrangements may occur more or less efficiently, depending on the TCR Tg and the selective advantage it confers at the DN and DP thymocyte stages and its survival adequacy in the peripheral environments. Second, the detection of T-cells bearing endogenous TCR in RAG-deficient Tg mice needs strong activation and selection either by allogeneic stimuli, as in the present study, or autoimmune reactivity [20] that do not often occur in classical protocols using specific immunization. Moreover, the robust T-cell activation in the allogeneic environment may have induced *RAG* gene re-expression by the donor T-cells [21]. By transferring sorted GFP^neg^ peripheral T-cells from Tg NG-BAC mice expressing GFP under the control of the RAG-2 regulatory sequences [22] into allogeneic hosts, we found that 6 weeks after transfer about 2–3% of the donor cells become GFP^+^ (Figure S1). *RAG* re-expression may have allowed new TCR chain recombination and editing [23], generating new chains that could readily associate with the available chain to promote further diversity. The immune system seems to use all possible means to avoid monoclonality (“horror monoclonicus”) [24] and ensure antigen-receptor diversity. These processes may have important implications as they allow modifications of the peripheral T cell repertoires with the arising of new specificities that can be selected and expanded by environmental antigens. Our findings illustrate that, in adoptive T cell transfer experiments using “monoclonal” T cells from RAG-deficient TCR Tg donors, monitoring the expression of the Tg TCR chains is an absolute requirement for accurate interpretation of the results particularly when these cells are transferred into “hostile” non-MHC compatible environments. They indicate that TCR Tg mice in triple TCRα, TCRδ and TCRβ KO mice may be ideal source of truly monoclonal T cells, as they would also ensure the absence of trans-lineage TCR expression [25].

## Materials and Methods

### Ethics Statement

Mice were cared for in accordance with Pasteur Institute guidelines in compliance with European animal welfare regulations, and all animal studies were approved by the Pasteur Institute Safety Committee in accordance with French and European guidelines.

### Mouse strains

The 5CC7 mice used in this study are Tg for the rearranged Vα11 and Vβ3 TCR chains specific for the COOH-terminal epitope of pigeon cytochrome c (PCC 88–104) in the context of I-E^k^. The 5CC7 Tg mice were made homozygous for RAG-2^0/0^ and the TCR Tg in a B10.A (H-2^a^) genetic background. This strain, referred to as B10.A/SgSnAi TCR–Cyt 5CC7-1 RAG-2^0/0^, can be purchased from the NIAID/Taconic Farms, Inc. Exchange. The H-2^b^ OT-1 RAG-1^0/0^ mice Tg for an anti-OVA H-2K^b^-restricted Vα2^+^Vβ5^+^ TCR [26] and the H-2^b^ aHY RAG-2^0/0^ mice Tg for an anti-HY H-2D^b^-restricted VαT3.70^+^Vβ8.2^+^ TCR were provided by the CDTA, CNRS, Orléans, France. RAG-2^0/0^γc^0/0^H-2^b^ and RAG-2^0/0^γc^0/0^H-2^a^ mice were kindly provided by J. Di Santo, Institut Pasteur, France and B. Stockinger, NIMR, UK respectively. Tg B6 mice carrying a BAC encoding GFP under the control of RAG-2 regulatory elements [22] were a gift from Dr. F. Huetz.

Genotyping of RAG-deficient strains were performed on tail DNA using a 35–40 cycle PCR. In each case, 3 primers were used enabling to detect both WT and KO allele: for RAG-1 KO mice: fwd5′CAATGTGCAGCTCAGCAAGAAACT3′–rvs5′TTCCAGACTCACTTCCTCATTGCA3′–neofwd5′GCATCGCCTTCTATCGCCTTCTTGACG3′ (WT band  = 421 pb; KO band  = 600 pb); for RAG-2 KO mice: fwd5′GGGAGGACACTCACTTGCCAGTA3′-rvs5′AGTCAGGAGTCTCCATCTCACTGA3′- neofwd5′CGGCCGGAGAACCTGCGTGCAA3′ (WT band  = 263 pb; KO band  = 350 pb).

### Adoptive transfers and cell phenotype analysis

10^6^ carboxyfluorescein succimyl esterate (CFSE)-labeled 5CC7 LN T-cells were injected i.v. into syngeneic RAG-2^0/0^γc^0/0^H-2^a^ and allogeneic RAG-2^0/0^γc^0/0^H-2^b^ hosts. For CFSE labeling, cells at 10^7^/ml in PBS were incubated with CFSE (Molecular Probes) at a final concentration of 5 µM for 12 min at RT and washed twice in RPMI 1640 containing L-alanyl-L-glutamine dipeptide and 10% FCS. In other experiments, H-2^b^ OT-1RAG-1^0/0^ TCR Tg or H-2^b^ aHYRAG-2^0/0^ TCR Tg LN T-cells were injected i.v. into syngeneic RAG-2^0/0^γc^0/0^H-2^b^ or allogeneic RAG-2^0/0^γc^0/0^H-2^a^ hosts. At different times after transfer, mice were sacrificed and spleen collected. After a blocking step with rat anti-CD16/32 mAbs (2.4G2), cells were stained using anti-CD3ε (145-2C11), anti-CD4 (RM4-5), anti-CD8α (53–6.7), anti-Vα11(RR8-1), anti-panβ (H57-597) and anti-Vβ3 (KJ25) mAbs from BD Pharmingen coupled with the appropriate FITC, PE, PerCP, APC, PECy7 dyes. Anti-Vα2, anti-Vβ5.1.2, anti-Vβ6, Vβ8.2 and anti-Vβ8.1.2.3 mAbs were also from BD Pharmingen. Dead cells and doublets were excluded. Ig control Abs are used for negative controls as well as stainings of non CD4 CD8 size comparable cells. All acquisitions and data analysis were performed with a FACScalibur or a FACSCanto (Becton Dickinson, San Jose, CA USA) interfaced to the Macintosh CellQuest or FlowJo software.

### CDR3 length analyses

Total RNA was extracted from 2–10.10^6^ thymus or spleen cells using TRIZOL according to the manufacturer procedure (Invitrogen). In some cases, total RNA was extracted from either CD4 or CD8 T-cells enriched by positive magnetic sorting using an AutoMACS (Milteny Biotec). cDNA was synthesized for 50 min at 42°C using superscript reverse-transcriptase (Invitrogen) in the presence of an inhibitor of RNAse (RNAsin, Promega). Protocols for TCR AV-AC (Vα-Cα) and BV-BC (Vβ-Cβ) CDR3 spectratyping have been described [11]. AC, AV, BC and BV primers were as described [11], except BV8.3 (TGCTGGCAACCTTCAAATAGGA) and BV13 (AGGCCTAAAGGAACTAACTCCAC). The nomenclature used follows that of Arden et al. [27]. PCR products were loaded on a 96-well ABI377 automated sequencer (Applied Biosystems, Foster City, CA) and separated according to their nucleotide length, forming a profile of peaks for each primer combination, spaced by 3 nucleotides as expected for in-frame transcripts. Each peak corresponds to a CDR3 length. The Immunoscope software [11] was used to obtain peak area and nucleotide length and CDR3 profile displays from sequencer raw data. As negative control for TCR recombination, mRNA from spleen cells of β-enhancer KO mice [12] was processed as the other samples and was found negative for the expression of TCRβ chains.

### Sequencing analyses

For single peak Vα and Vβ profiles, direct sequencing was performed with AV and AC, and BV and BC primers following the recommendations of the BigDye Terminator v1.1 Cycle Sequencing Ready Reaction kit (Applied Biosystems). PCR products were then incubated with 0.5 U of shrimp alkaline phosphatase and 5 U of exonuclease I (GE Healthcare) at 37°C for 40 min, followed by 20 min at 80°C. DNA alignments were performed using the GCG package (Genetics Computer Group, Accelrys, Cambridge, U.K.). For multi-peak Vα and Vβ profiles, AV-AC and BV-BC PCR products were cloned using the TOPO TA cloning kit (Invitrogen) following the manufacturer's instructions. Plasmid DNA from individual colonies was amplified with the same primers and sequenced, as above.

## Supporting Information

Figure S1 RAG-2-re-expression in OT-1 TCR Tg T cells.
(0.14 MB TIF)
