# Supplementary figures and images for: Endogenous TCR Recombination in TCR Tg Single RAG-Deficient Mice Uncovered by Robust In Vivo T Cell Activation and Selection
Source: PLoS One. 2010 Apr 29;5(4):e10238. doi: 10.1371/journal.pone.0010238 (PMC2861594; doi:10.1371/journal.pone.0010238)

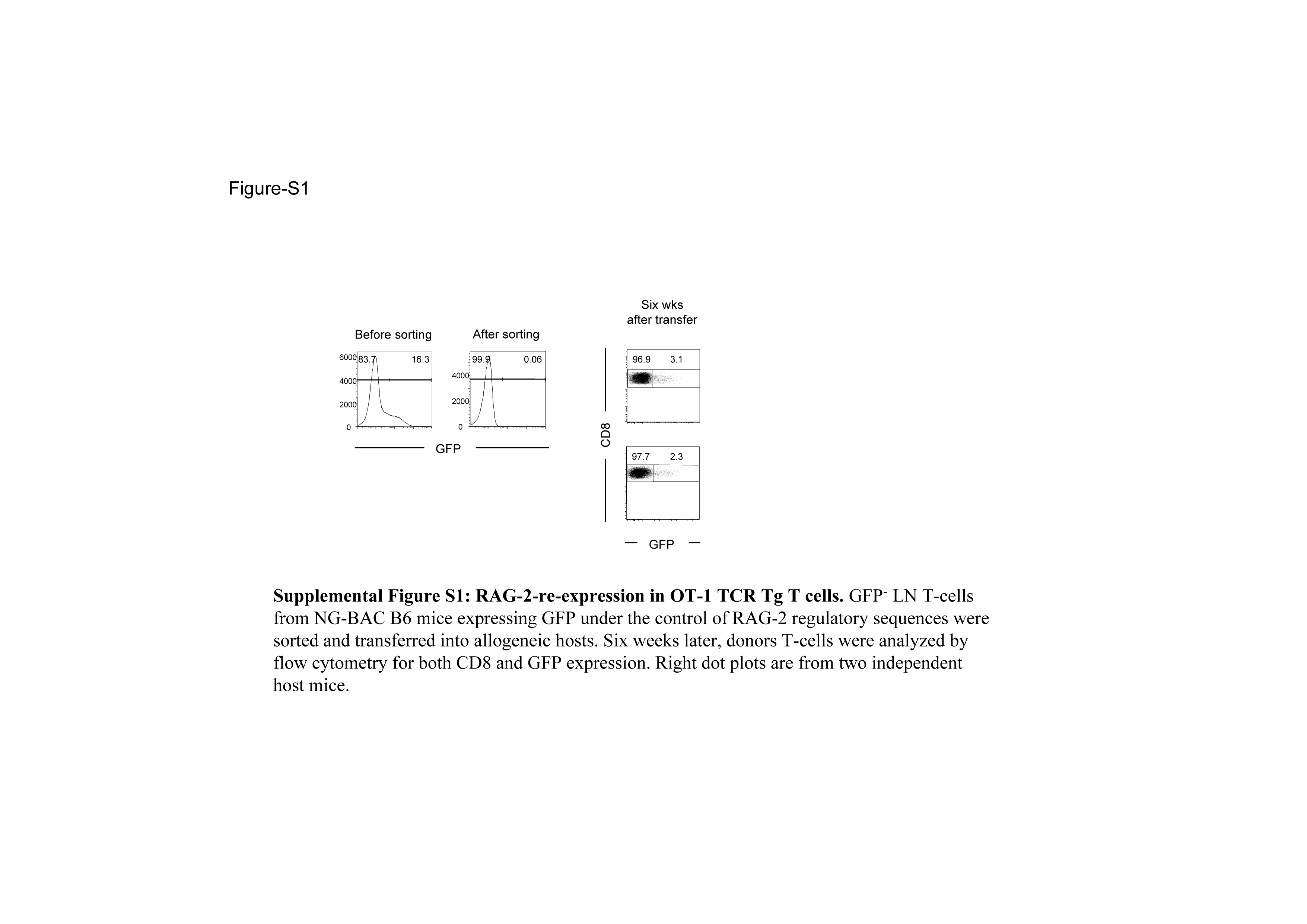

Supplement: Figure S1 — RAG-2-re-expression in OT-1 TCR Tg T cells. (0.14 MB TIF) [file pone.0010238.s001.tif]
